# Coupled blue and red light-emitting diodes therapy efficacy in patients with rosacea: two case reports 

**DOI:** 10.1186/s13256-019-2339-6

**Published:** 2020-01-28

**Authors:** Elisabetta Sorbellini, Maria Pia De Padova, Fabio Rinaldi

**Affiliations:** 1 International Hair Research Foundation (IHRF), Milan, Italy; 2 Dermatology, Nigrisoli Private Hospital, Bologna, Italy

**Keywords:** Rosacea, Light-emitting diodes, LED, Photodynamic therapy

## Abstract

**Background:**

Rosacea is a common inflammatory skin condition affecting approximately 5% of the world population. Therapeutic approaches to rosacea are focused on symptom suppression by means of anti-inflammatory agents. More recently, photodynamic therapy, especially light-emitting diodes, has been introduced as a valid alternative to conventional therapy.

**Case presentation:**

In the present work, we reported the efficacy and safety of light-emitting diodes therapy combining blue (480 nm) and red (650 nm) light for the treatment of two patients with papulopustular rosacea: a 22-year-old Caucasian woman and a 68-year-old Caucasian man.

**Conclusions:**

This kind of treatment could represent an effective, safer, and well-tolerated approach for the treatment of such conditions.

## Background

Acne rosacea, usually referred to as rosacea, is a common inflammatory skin condition affecting mainly the central face [1]. The term was introduced by Thomas Bateman in the nineteenth century as an acne variety [2]. Its typical manifestations are generalized erythema, telangiectasia, and edema, then papules and pustules or a combination of all [3, 4].

In 2004, the National Rosacea Society (NRS) Expert Committee published a report on the classification and staging of rosacea that defined the criteria for rosacea classification and grading according to primary and secondary descriptors [5]. Four subtypes of rosacea can be recognized on the basis of different morphological characteristics: erythematotelangiectatic, papulopustular, phymatous, and ocular [5, 6]. The erythematotelangiectatic subtype is the most common one followed by papulopustular, phymatous and ocular types which are reported as less common [7]. Data from clinical practice show that patients often can harbor more than one rosacea subtype [7]; for this reason, incidence and prevalence evaluation is not simple. The latest data population, based on published data, refers to an incidence of 1.65 per 1000 persons per year [8] indicating approximately 5.46% of the worldwide population [9]. A stronger predominance for females was found for erythematotelangiectatic and papulopustular subtypes with a diagnosis, usually, after the fourth decade of life [8, 10].

The exact pathogenesis of rosacea remains unclear but the involvement of several external or endogenous factors is reported [1, 11]. In fact, recent findings highlighted the role of predisposing factors such as genetic predisposition and association with other diseases [12]. Microbial stimuli, especially colonization, ultraviolet (UV) radiation, stress, and environmental changes are also recognized as triggering factors both for the development and worsening of rosacea [12–14]. Therefore, dysregulation of innate immunity via the expression of higher amounts of toll-like receptor 2 (TLR2) in the skin [15] and augmentation of the inflammatory cascade have been reported [16] as abnormal expression of cathelicidin antimicrobial peptides [17].

More recently, rosacea and other skin diseases such as psoriasis and atopic dermatitis have been linked to intestinal dysbiosis [18, 19]. Authors reported the role of intestinal dysbiosis in promoting inflammation and impairment of normal lymphocyte function, potentially perpetuating chronic, low-grade inflammation [20]. Therefore, the potential role of microorganisms in the pathogenesis of rosacea has been hypothesized [21]. Parodi and colleagues [22] reported a higher incidence of small intestinal bacterial overgrowth (SIBO) when patients with rosacea were compared to controls. Most interesting, microbial unbalancing of the skin microbiota on the skin has been linked to rosacea clinical manifestations [23], even though the direct correlation between microbiota composition on the skin and the incidence of the pathology is still under investigation.

More recently, in a NRS-supported study in twins, Zaidi and colleagues [24] reported the first evidence highlighting the correlation between the severity of rosacea and microbial dysbiosis on the skin, but further study is needed to determine the species involved.

Historically, therapeutic approaches to rosacea focused on symptom suppression by means of anti-inflammatory agents such as doxycycline [25–27], metronidazole [28], topical azelaic acid [11, 29], sodium sulfacetamide [11, 30], and calcineurin inhibitors [31]. The use of serine protease inhibitors is to be considered an emerging therapy in rosacea [32].

Several concerns surround the use of tetracyclines, especially as long-term treatment is often necessary. Although it is commonly prescribed at a sub-antimicrobial dose, gastrointestinal side effects and photosensitivity are not uncommon and the risk of antimicrobial resistance increases with higher doses [33, 34].

Although not yet approved for the treatment of rosacea, efficacy of a low dose of isotretinoin has been reported in patients with papulopustular rosacea subtype [35].

More recently, photodynamic therapy (PDT), especially light-emitting diodes (LED), has been introduced as a valid alternative to conventional therapy [36]. A few *in vitro* studies [37, 38] and a published *in vivo* study on patients with papulopustular rosacea with methyl ester aminolevulinate (MAL) coupled with PDT [39], reported efficacy of LEDs for treatment of rosacea.

## Case presentations

### Case report 1

A 22-year-old Caucasian woman presented to a dermatological clinic with a 5-year history of pink eruptions on her nose. She also reported a burning sensation. She was diagnosed as having papulopustular rosacea subtype, moderate grade, according to the classification and staging of rosacea developed by the NRS Expert Committee [5]. In the previous 2 years she was treated with two cycles of orally administered tetracycline (Lymecycline), 300 mg per day, for 12 weeks. Systemic therapy was associated with metronidazole cream 1% for cycles of 6 months. In the last 6 months before the visit, she also submitted to 40% pyruvic acid peeling every 25 days, with poor response and continuous relapses. A combined and sequential plan of blue (480 nm ± 15 nm, 300 J/minute) and red (650 ± 15 nm, 100 J/minute) LED therapy regimen was planned twice a week for a total of ten sessions. A quasi-monochromatic 120 LED system (Dermodinamica® instrument, ELISOR Srl, Milan, Italy) was used for 15 minutes (each wavelength).

### Case report 2

A 68-year-old Caucasian man presented with a 7-year history of papulopustular rosacea, moderate grade [5], which extended over the entire surface of his face. He had experienced extended relapses on his face once a year in the past 6–7 years. He was previously treated with two cycles of Lymecycline (tetracycline) at 300 mg per day or azithromycin every 2 weeks in combination with 0.75% topical metronidazole. He was submitted to LED therapy twice a week for a total of ten sessions. Blue (480 nm ± 15 nm, 300 J/minute) and red (650 ± 15 nm, 300 J/minute) were sequentially irradiated for 15 minutes by means of LED system Dermodinamica® (ELISOR Srl, Milan, Italy). The therapy was coupled with topical 15% azelaic acid.

### Outcome and follow-up

**Fig. 1:**
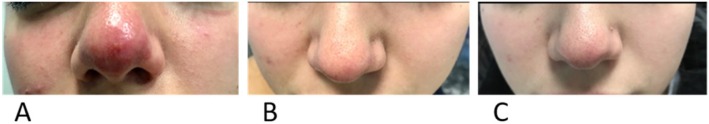
Papulopustular rosacea on the nose of case report 1 at the base time (**a**), after five sessions (**b**), and after ten sessions (**c**) with coupled blue (480 nm) and red (650 nm) light-emitting diodes therapy

**Fig. 2:**
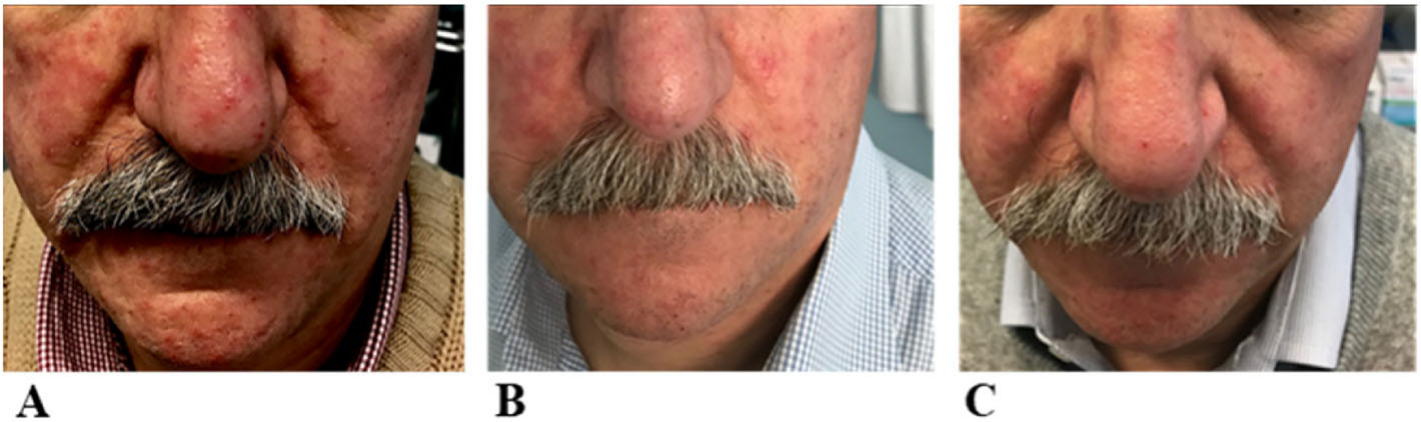
Papulopustular rosacea with erythema and telangiectasias on the glabella, forehead, nose, cheeks, and chin of case report 2 at the base time (**a**), after five sessions (**b**), and after ten sessions (**c**) with coupled blue (480 nm) and red (650 nm) light-emitting diodes therapy

Erythema, burning sensation, and itching were assessed using a visual scale grading (0 = no symptoms, 4 = very severe). Erythema and papules were subjectively assessed by the dermatologist, whereas the intensity of itch and burning sensations was expressed by our patients. A good response was obtained for both patients after ten treatments with LEDs. Both patients reported a reduction of symptoms such as burning and itching. Also, a reduction of erythema and papules was observed after five sessions of LED therapy (Figs. 1b and 2b). Further improvement was observed at the end of treatment: ten sessions of LED therapy (Figs. 1c and 2c).

## Discussion and Conclusions

Several therapeutic approaches are currently available for treating rosacea and they are mainly aimed at controlling disease symptoms [40, 41]. The therapeutic plan has to be adapted to the rosacea subtype and tailored according to the dominant manifestations of the patient [32, 35]. In general, the reduction of oral therapy in favor of topical or physical therapy is desirable in order to reduce side effects for patients and increasing the safety of treatment [5, 32].

The therapeutic approach described in this report aims at reporting the efficacy and safety of combined blue (480 nm ± 15 nm) and red (650 ± 15 nm) LED light-based therapy in patients affected by rosacea.

Previous research reported the efficacy of red and blue light coupled for the treatment of mild to moderate acne lesions [42, 43]. Blue light (400–470 nm), due to its lower penetration, is useful in such skin conditions related to the epidermis layer of the skin [44]; therefore, it is also able to interfere with human sebocytes proliferation [45]. On the other hand, red light (630 nm) is reported to have a significant effect on sebum production [46, 47]. The benefits deriving from PDT using LEDs are not limited to its efficacy but are also related to its safety and tolerance by patients; therefore, its advantages can be extended to a broad range of dermatological conditions [48, 49].

In fact, PDT is routinely used by dermatologists in the treatment of moderate to severe acne vulgaris [50, 51] and perioral dermatitis [18]. Rosacea shares several features with other dermatological diseases, especially acne. In patients with acne, PDT has been supposed to act via modulation of the functionality of the pilosebaceous unit and this could probably also be applied to rosacea.

Previous to our work, several authors reported the efficacy of PDT therapy on patients with rosacea [36, 52–54]. Moreover, an *in vitro* study on rosacea-like mouse skin [55] reported the efficacy of LED at 630 and 940 nm on the down-regulation of key inflammatory mediators of rosacea, such as cathelicidin (LL-37), TLR2, and kallikreins (KLKs). These results are in line with reported evidence on the efficacy of LED therapy also to interact with the host immune system. LEDs may also interact with skin microbiome [56–58] and this could also have as significant an impact on the etiopathogenesis of rosacea as on immune response modulation. A deeper knowledge of the implications of both gut and skin microbiome in rosacea is still needed; our recent research is aimed at evaluating the real effect of blue and red light LEDs on skin microflora in patients with rosacea and patients with acne.

In addition, the safety deriving from the use of LED devices encourages their ever-increasing use for the treatment of many dermatological conditions, including rosacea.

Nowadays, the treatment of patients with rosacea still represents a challenge for dermatologists. Conventional treatment of rosacea is either ineffective or results in the dissatisfaction of patients due to the need for continuous treatment.

The case reports presented in the current work show, for the first time, the usefulness of LED therapy combining blue and red light benefits for the treatment of patients with rosacea. This kind of treatment could represent an effective, safer, and well-tolerated approach for the treatment of such kinds of condition.

## Data Availability

Not applicable.
